# First Commencement of the Howard University, Washington, D. C.

**Published:** 1871-02

**Authors:** 


### First Commencement of the Howard University, Washington, D. C.

We have received a very polite invitation to attend the first commencement of the Howard University, to be held March 15th. The charge to the graduate is to be given by Prof. P. H. Strong, M. D. The address is by the president of the university. The following gentlemen constitute the graduating class: William W. Bennett, George W. Brooks, Danforth B. Nichols, J. A. Sladen.

